# Co-Regulation of NF-κB and Inflammasome-Mediated Inflammatory Responses by Myxoma Virus Pyrin Domain-Containing Protein M013

**DOI:** 10.1371/journal.ppat.1000635

**Published:** 2009-10-23

**Authors:** Masmudur M. Rahman, Mohamed R. Mohamed, Manbok Kim, Sherin Smallwood, Grant McFadden

**Affiliations:** Department of Molecular Genetics and Microbiology, College of Medicine, University of Florida, Gainesville, Florida, United States of America; Ludwig-Maximilians-Universität München, Germany

## Abstract

NF-κB and inflammasomes both play central roles in orchestrating anti-pathogen responses by rapidly inducing a variety of early-response cytokines and chemokines following infection. Myxoma virus (MYXV), a pathogenic poxvirus of rabbits, encodes a member of the cellular pyrin domain (PYD) superfamily, called M013. The viral M013 protein was previously shown to bind host ASC-1 protein and inhibit the cellular inflammasome complex that regulates the activation and secretion of caspase 1-regulated cytokines such as IL-1β and IL-18. Here, we report that human THP-1 monocytic cells infected with a MYXV construct deleted for the M013L gene (vMyxM013-KO), in stark contrast to the parental MYXV, rapidly induce high levels of secreted pro-inflammatory cytokines like TNF, IL-6, and MCP-1, all of which are regulated by NF-κB. The induction of these NF-κB regulated cytokines following infection with vMyxM013-KO was also confirmed *in vivo* using THP-1 derived xenografts in NOD-SCID mice. vMyxM013-KO virus infection specifically induced the rapid phosphorylation of IKK and degradation of IκBα, which was followed by nuclear translocation of NF-κB/p65. Even in the absence of virus infection, transiently expressed M013 protein alone inhibited cellular NF-κB-mediated reporter gene expression and nuclear translocation of NF-κB/p65. Using protein/protein interaction analysis, we show that M013 protein also binds directly with cellular NF-κB1, suggesting a direct physical and functional linkage between NF-κB1 and ASC-1. We further demonstrate that inhibition of the inflammasome with a caspase-1 inhibitor did not prevent the induction of NF-κB regulated cytokines following infection with vMyxM013-KO virus, but did block the activation of IL-1β. Thus, the poxviral M013 inhibitor exerts a dual immuno-subversive role in the simultaneous co-regulation of both the cellular inflammasome complex and NF-κB-mediated pro-inflammatory responses.

## Author Summary

Myxoma virus (MYXV), a rabbit-specific poxvirus pathogen, encodes diverse immunomodulatory proteins that can collectively overcome essentially all of the host immune defenses. MYXV-encoded protein M013, a member of the cellular PYRIN domain-containing superfamily of proteins, was previously shown to be important for virus virulence by blocking inflammasome regulated pro-inflammatory cytokine secretion. Here, we report that, in addition to targeting the ASC-1 protein of the host cell inflammasome complex, M013 also blocks activation of NF-κB signaling pathway by interacting with NF-κB1 and preventing nuclear translocation of the transcription factor RelA/p65. MYXV virus lacking a functional M013L gene (vMyxM013-KO) induced the early activation of NF-κB signaling pathway in human monocytic cells, causing the secretion of antiviral pro-inflammatory cytokines *in vitro* and *in vivo*. These results demonstrate that MYXV protein M013 is a multifunctional immunosubversive protein that co-regulates both the inflammasome complex and the NF-κB signaling pathway simultaneously.

## Introduction

The nuclear factor κB (NF-κB) family comprise a set of related transcription factors that regulate multiple cellular pathways associated with immune responses, inflammation, apoptosis, cell growth and survival [1],[2]. The mammalian NF-κB family members include NF-κB1 (p105 and p50), NF-κB2 (p100 and p52), RelA (p65), RelB and c-Rel, which become activated by upstream signals from diverse immune receptors, such as ligand-triggered Toll-like receptors (TLRs), interleukin-1 receptor (IL-1R), tumor necrosis factor receptor (TNFR) and antigen receptors [3],[4]. However, their activation is tightly regulated by another family of proteins known as inhibitors of NF-κB (IκBs), which are also associated with the regulation of these diverse cellular processes [5]. In unstimulated cells, NF-κB proteins remain inactive in the cytoplasm, usually as homodimers or as heterodimers with RelA, RelB or c-Rel and complexed with the inhibitory IκBs. The IκBs are in turn regulated by another group of regulatory proteins called IκB kinases (IKKs). The IKK complex is composed of two catalysis subunits (IKKα and IKKβ) and a regulatory subunit IKKγ, or NEMO (NF-κB essential modulator) [6],[7],[8]. Upon ligand engagement, the receptors initiate signal transduction events that lead to the activation of IKK complex by activated cellular protein kinases like NF-κB-inducing kinase (NIK), mitogen-activated protein/extracellular signal-regulated kinase1 (MEKK1), transforming growth factor-β (TGFβ)- activated kinase 1 (TAK1), MEKK2 or MEKK3. Activated IKK phosphorylates IκBα using specific serine residue within the IκBα proteins, triggering their ubiquitination via ubiquitin ligase [9]. The IκBα protein is then degraded by the 26S proteasome, allowing the release and translocation of the active NF-κB dimer into the nucleus to upregulate targeted gene transcription events [10],[11].

Subversion of the host cell NF-κB signaling is a key strategy adapted by many pathogens because this pathway regulates the expression of multiple cellular host response proteins such as anti-viral cytokines, chemokines and the presentation of viral antigens to cytotoxic T lymphocytes [12],[13],[14]. Amongst viruses, members of the poxvirus family are known to target and downregulate NF-κB signaling in multiple ways. One strategy is to directly block the primary immune ligands, particularly cytokines, chemokines or IFNs, and thus prevent binding and activation of their cognate receptors. In many cases these viral inhibitors (called viroceptors) are secreted homologs of corresponding cellular receptors [15],[16]. Another strategy is to deploy viral proteins to target the components of the intracellular NF-κB signaling pathways [17]. For example, vaccinia virus (VACV), the prototypic member of the *poxviridae* family and the most extensively studied poxvirus, encodes multiple proteins to inhibit the intracellular NF-κB pathway at different steps of the signaling cascade. For example, VACV-encoded proteins A52R and A46R inhibit the interleukin 1 receptor (IL-1R) and toll-like receptor (TLR) signaling pathway linked to the NF-κB complex [18],[19]. A52R inhibits NF-κB activation by interaction with interleukin 1 receptor-associated kinase 2 (IRAK2) and tumor necrosis factor receptor associated factor (TRAF6) [20]. B14R was recently shown to bind the IKK complex and prevent phosphorylation of the IKKβ activation loop [21]. Another VACV protein, N1L, also targets the IKK complex and interferes with both NF-κB and interferon regulatory factor 3 (IRF3) signaling [22]. Among the other VACV proteins, K1L inhibits NF-κB activation by blocking degradation of IκBα [23] while M2L downregulates ERK-mediated NF-κB induction in infected cells [24]. Another poxvirus, molluscum contagiosum virus (MOCV), also encodes multiple proteins, for example MC159 and MC160, which regulate the NF-κB signaling pathways [25],[26],[27].

Myxoma virus (MYXV) is a member of the leporipoxvirus genus of the *poxviridae* family and causes lethal disease myxomatosis in European rabbits (*Oryctolagus cuniculus*) [28]. MYXV encodes diverse secreted and intracellular immunomodulatory proteins to overcome host immune defenses [29]. However, unlike most of the members of the orthopoxvirus genus, MYXV does not express any predicted secreted proteins that directly bind and neutralize pro-inflammatory cytokines such as IL-1β and IL-18, which are regulated by a series of cellular multi-protein complexes collectively termed inflammasomes [30],[31],[32]. Instead, the MYXV-encoded protein M013, which was first identified as a member of the PYRIN domain (PYD) superfamily, was shown to function as an intracellular inhibitor of inflammasome activation [33]. The cellular PYD-containing proteins mediate protein-protein interactions with components of signaling pathways involved in the regulation of apoptosis, NF-κB activation and pro-inflammatory cytokine production [34],[35]. Our initial study showed that a MYXV construct deleted for M013L (vMyxM013-KO) was significantly attenuated in host rabbits because of decreased virus dissemination and enhanced inflammatory responses at the tissue sites of virus infection [33]. The vMyxM013-KO virus was unable to productively infect rabbit monocytes or lymphocytes due to an abortive phenotype, compared to parental MYXV. M013 protein was also shown to directly interact with apoptosis-associated speck-like protein containing CARD-1 (ASC-1), a component of the cellular inflammasome complex and inhibited caspase-1 activation and the processing of pro-inflammatory cytokines IL-1β and IL-18 [33].

In the present study, we report that infection of human THP-1 monocytic cells with the vMyxM013-KO virus, unlike the parental MYXV, unexpectedly induced rapid and dramatic secretion of diverse pro-inflammatory cytokines such as TNF, IL-6 and MCP-1, all of which are regulated by NF-κB. THP-1 cells infected with vMyxM013-KO virus, but not the parental MYXV, activated the IKK kinases and degradation of IκBα at very early time of infection, resulting in the activation and nuclear translocation of NF-κB. We further demonstrate that the expressed M013 protein alone interacts directly not only with ASC-1, but also with host NF-κB1 and inhibits the translocation of p65 to the nucleus. We conclude that the viral PYD-containing M013 protein can simultaneously bind and co-regulate key components from both the inflammasome complex and NF-κB-mediated signaling pathways.

## Materials and Methods

### Reagents and antibodies

Recombinant TNF was purchased from Biosource. Rabbit polyclonal antibodies (pAb) for IκBα, phospho-IκBα, p65, NF-κB1, IKKα/β, phospho-IKKα/β, were purchased from Cell Signaling Technology. HRP-conjugated goat anti-rabbit and anti-mouse IgG antibodies were purchased from Jackson Lab. Phorbol-12-myristate-13-acetate (PMA), LPS (lipopolysaccharide), and caspase-1 inhibitor zVAD-fmk were purchased from Sigma. ERK1/2 inhibitor U0126 and PI3kinase inhibitor LY294002 were purchased from Cell Signaling technology.

### Plasmid construction

Cloning strategies used are based on the Gateway cloning technology (Invitrogen). The M013L ORF of myxoma virus was PCR amplified from the virus genome using Pfu Ultra polymerase (Stratagene) and cloned in the Gateway entry vector pDONR221 (Invitrogen) using BP Clonase enzyme mix (Invitrogen). M013L ORF lacking the PYD (1–81 amino acids) was constructed by PCR amplification using appropriate primers and cloned in the entry vector pDONR221. Both human RelA and NF-κB1 cDNAs (Open Biosystems) were PCR amplified and cloned in pDONR222 vector (Invitrogen). The resultant entry clones were subsequently cloned in expression vectors pANT7_cGST, pANT7_nHA [36], pcDNA3.1MycHis and pDEST40 (Invitrogen) using the LR Clonase II enzyme mix (Invitrogen).

### Cell lines and cell culture

BGMK, 293T, BSRT7/5 (BHK cells expressing T7 polymerase) [37] cells were cultured in DMEM supplemented with 10% heat-inactivated fetal bovine serum (FBS), 2 mM L-glutamine, 100 U/ml penicillin and 100 µg/ml streptomycin (pen-strep). THP1 cell line was cultured in RPMI 1640 medium (Lonza) supplemented with 10% FBS and pen-strep. For differentiation, THP1 cells were stimulated for 18 h (hour) with 100 ng/ml PMA.

### Viral preparation

Construction of a wild-type myxoma virus (vMyx-gfp) that expresses a GFP cassette under the control of a synthetic VACV early/late promoter was described previously [38]. Construction of the vMyxM013-KO virus was previously described [33]. Viruses were purified by centrifugation through a sucrose cushion and two successive sucrose gradient sedimentations as described previously [39].

### Transfections and luciferase assays

HeLa cells (1×10^4^) were seeded at 50% confluence onto 96-well plates the day before being transfected. Cells were transfected according to the manufacturer's protocol using 0.5 µl Lipofectamine 2000 reagent (Invitrogen) and 0.2 µg DNA, in serum-free minimum essential medium (MEM), per each well. The DNA was a 1∶1∶1 mix of NF-κB reporter vector to constitutive expression vector to viral protein expression vector. The NF-κB reporter vector used was the Stratagene (La Jolla, CA) pNFκB-Luc plasmid, which gives inducible NF-κB-dependent expression of firefly (*Photinus pyralis*) luciferase driven by a synthetic promoter comprising a TATA box preceded by five direct repeats of the sequence 59-TGGGGACTTTCCGC-39 containing the NF-κB binding element first identified in the kappa light chain gene enhancer [40]. The constitutive expression vector used was the Promega (Madison, WI) pUCbased pRL-TK vector, which gives low-level constitutive expression of sea pansy (*Renilla reniformis*) luciferase from the promoter of the herpes virus thymidine kinase gene. M013L ORF was cloned in pcDNA3.1MycHis vector (Invitrogen) that gives a constitutive level of protein expression from the CMV promoter [33]. At 6 h post-transfection, FBS was added to the medium for a final concentration of 10%. At 48 h post-transfection, the cells were treated with 20 ng/ml TNF for 6 h. To determine luciferase values, the Promega Dual-Luciferase Reporter Assay System was used according to the manufacturer's instructions, with slight modifications. Briefly, the medium was removed and the cells were washed once with 50 µl PBS and the cells were lysed in 20 µl of 1× passive lysis buffer (Promega) for 15 min at room temperature. The lysates were then analyzed for firefly and sea pansy luciferase activity. For luciferase value determination, 100 µl of luciferase assay reagent II (Promega) were added to the cell lysate and analyzed for firefly luciferase activity using the Appliskan multimode microplate reader (Thermo Scientific). Then 100 µl of Stop & Glo reagent (Promega) were added for determination of sea pansy luciferase activity. The relative fold changes were determined by normalizing the ratios of firefly to sea pansy luciferase activities in each transfected cell group to the value obtained for non-transfected cells.

### Western blotting

For detection of protein, cells were harvested at different time points, washed with PBS and stored at −80°C or processed immediately with RIPA lysis buffer (50 mM Tris, 150 mM NaCl, 0.1% SDS, 0.5% sodium deoxycholate, 1% NP40, 1 mM PMSF, protease inhibitor cocktail (Roche)). Protein samples were separated on SDS-PAGE gels and transferred to PVDF membrane (GE Healthcare) using a semidry transfer apparatus (Fisher). Membranes were blocked in TBST (20 mM Tris, 150 mM NaCl, 0.1% Tween-20 pH 7.6) containing 5% non-fat dry milk for 1 hr at room temperature and then incubated overnight with primary antibody at 4°C. The membranes were washed three times, 15 minutes each with TBST and incubated with HRP-conjugated goat-anti-mouse(1∶5000) or goat-anti-rabbit (1∶5000) secondary antibody in TBST containing 5% non-fat dry milk for 1 hour at room temperature with gentle agitation. The membranes were washed three times, 15 minutes each with TBST, and the signal was detected following the application of chemiluminescence substrate (Pierce) and exposure to X-ray film (Eastman Kodak).

### Quantification of cytokine secretion by ELISA

THP-1 cells were plated in multi-well plates in the presence of PMA (100 ng/ml) for 12–18 h. The following day, media were replaced with fresh media and the cells were either infected with WT myxoma and vMyxM013-KO viruses (at MOI of 3) or treated with LPS and the supernatants were collected at different time points. Whenever mentioned, inhibitors were added one hour before viral infection. The level of secreted cytokines TNF, IL-1β, IL-6, IL-12 and MCP-1 were determined using ELISA assay kits (eBioscience) following manufacturer protocol.

### In vitro transcription/translation of plasmid constructs

The rabbit reticulolysate coupled transcription and translation (TnT) system (Promega) was used according to manufacturer's protocol for expression of proteins in vitro. Protein expression was confirmed by running 2.5 µl of the TnT reaction on SDS-PAGE gels followed by western blot analysis using anti-GST antibody (Neomarkers) for the GST-tagged protein and anti-HA antibody (Santa Cruz) for the HA-tagged protein.

### AlphaScreen protein interaction assay

The Amplified Luminescent Proximity Homogeneous Assay (AlphaScreen™; PerkinElmer) [41],[42] is a bead-based technology for screening biomolecular interactions in a microplate format [43]. The assay was performed in 384-well white opaque optiplates (Perkin Elmer) in a total volume of 25 µl. BSR T7/5 cells were used for expression of tested proteins. Cells were seeded onto 24-well plates one day before transfection. Cells were then transfected according to the manufacturer's protocol using 2 µl Lipofectamine 2000 reagent (Gibco BRL) and 0.8 µg total DNA (equal amounts of host and viral DNA), in serum-free MEM, per each well. The expression vectors used were either the pcDNA3.1MycHis plasmid (Invitrogen), for the host proteins, or the pANT7_cGST plasmid (Harvard Institute of Proteomics), for the viral protein. Expression from the pcDNA3.1MycHis plasmid is under the control of either CMV or T7 RNA polymerase promoter and the expressed proteins are fused at their C-terminal with both Myc and His tags. On the other hand, expression from the pANT7_cGST plasmid is under the control of T7 RNA polymerase promoter and expressed proteins are fused at their C-terminal with a glutathione-S-transferase (GST) tag. Forty eight hours post transfection, cells were collected in 500 µl PBS, pelleted and then lysed by suspending in 20 µl lysis buffer (100 mM NaCl, 100 mM Tris, pH 8.0, 0.5% NP-40, containing 25 µl/ml Roche complete protease inhibitor). The cellular extracts were then separated following centrifugation for 5 min at 13,000 rpm.

All dilutions were made in phosphate buffered saline (PBS; 3.2 mM Na_2_HPO_4_, 0.5 mM KH_2_PO_4_, 1.3 mM KCl, 135 mM NaCl, pH 7.4) containing 0.1% bovine serum albumin (BSA). Five µl of anti-GST acceptor beads (PerkinElmer, 6760603C), diluted 50-fold (20 µg/ml final concentration of beads in assay) in 0.1% BSA/PBS assay buffer, and 5 µl of cell extract were mixed to a final volume of 20 µl with 0.1% BSA/PBS assay buffer, and incubated at room temperature for 1.5 hour. Subsequently, 5 µl of Nickel Chelate donor beads (PerkinElmer, AS101D), diluted 50-fold in 0.1% BSA/PBS assay buffer (20 µg/ml final concentration of beads in assay), were added to a final volume of 25 µl and incubation was continued for another 1.5 hour at room temperature. All additions and incubations were performed under subdued lighting conditions due to the photosensitivity of the beads. The plate was then read on an EnVision multiwell plate reader (PerkinElmer).

### Detection of protein-protein interaction using ELISA

A modified Enzyme-linked immunosorbent assay (ELISA) was used for *in-vitro* protein-protein interaction studies. The 384-well ELISA plates were coated with a rabbit polyclonal anti-GST antibody (Neomarkers), diluted 1 to 400 in coating buffer (0.138 M NaCl, 0.0027 M KCl, pH 7.4), overnight at 4°C in 25 µl volume. Following incubation, the antibody was removed and wells were blocked overnight with 5% non-fat milk in PBS. Blocking buffer was removed and wells were washed 5 times with 100 µl washing buffer (PBS containing 1% BSA and 0.05% Tween 20). Both viral and host proteins were expressed, either individually or in combination, using the TnT *in vitro* expression system following manufacturer's protocol. Either 1.25 µl or 2.5 µl (for individually expressed or co-expressed proteins, respectively) of each TnT reaction was then applied, per well, to the anti-GST antibody coated plate and incubated for 2 hr at room temperature. The wells were then washed 5 times with washing buffer. Assembly of the protein complex in the wells was then assessed through the incubation of HRP-conjugated rat anti-HA antibody (1∶500 dilution) (Roche) in 25 µl volume for 2 hr at room temperature. The unbound antibody was removed by washing the wells 5 times with washing buffer. Binding of the second antibody (HRP-conjugated anti-HA) to the protein complex was then detected by applying 50 µl of TMB substrate. The reaction was stopped by adding 25 µl of 2 N H_2_SO_4_ to each well. The plate was read at 450 nm using a multi-well plate reader.

### Immunofluorescence confocal microscopy

HeLa cells (3×10^4^) plated on coverslips were either mock transfected or transiently transfected with 2 µg of pcDNA3.1M013L or pDEST40M013ΔPYD DNA. Cells were mock treated or treated with 20 ng/ml TNF for 30 min at 37°C at 24 hr post transfection. Coverslips were then washed with PBS and fixed with 2% paraformaldehyde (Sigma) for 12 minutes, permeabilized with 1% NP40 (Sigma) and blocked with 3% BSA in PBS. Coverslips were incubated with both rabbit anti- p65 (sc-372; Santa Cruz) and mouse anti-Myc (sc-40; Santa Cruz) or mouse anti-V5 (Invitrogen) diluted 1∶100 in PBS containing 3% BSA for 30 minutes at 37°C. Coverslips were incubated in these secondary antibodies: goat anti-rabbit-Texas-Red and goat anti-mouse- fluorescein isothiocyanate diluted at 1∶750 in PBS containing 3% BSA for 30 minutes at 37°C in the dark. Following staining, coverslips were mounted on microscope slides with 7.5 µl Vecta-shield mounting media with DAPI (4′,6 diamidino-2-phenylindole; Vector Laboratories, Burlingame, CA, USA) for visualization of nuclei. Cells were visualized using the 40X objective of an Olympus DSU-IX81 Spinning Disc Confocal/Deconvolution fluorescent microscope.

### *In vivo* infections of THP-1 tumor xenografts

Xenografted THP-1 tumors were generated by implanting THP-1 cells (5×10^6^ in 100 µl PBS) subcutaneously (s.c) on the left hind leg of 6–8 week old female NOD-SCID mice (Charles River). Tumor growth was monitored every other day. On day 6 after tumor cell implantation, a single dose of MYXV or vMyxM013-KO (1×10^8^ ffu virus in 100 µl volume) was injected directly in the tumor. The animals of the control group were injected with PBS into the tumor. After 48 h following virus injection, the animals were sacrificed and both blood and tumor tissues (suspended in PBS) were collected. The levels of secreted human cytokines in serum and tumor tissues were determined by ELISA as described before. All animal studies were performed in compliance with the regulations of the University of Florida Animal Care Services in accordance with the guidelines set by the Association for Assessment and Accreditation of Laboratory Animal Care.

## Results

### Induction of an early pro-inflammatory cytokine secretion by vMyxM013-KO virus infection of THP-1 cells

The vMyxM013-KO virus was markedly attenuated in infected rabbits due to enhanced inflammatory responses at sites of infection, suggesting that M013 was involved in regulating host inflammatory responses to the virus infection [33]. Since many MYXV-encoded modulators are able to recognize and inhibit host proteins from a wide variety of species, particularly if the host protein targets are well conserved, this phenomenon was further investigated in cultured human monocytic THP-1 cells, where the secretion of the inflammasome-controlled cytokines IL-1β and IL-18 was previously shown to be inhibited by M013 protein. The PYD domain of M013 was critical for the protein/protein interaction with host ASC-1 protein, the common adapter protein of inflammasome complexes, and prevented caspase-1-mediated activation and secretion of these cytokines in response to MYXV infection [33]. It has been reported that some cellular PYD-containing regulatory proteins can co-modulate the function of both inflammasomes and NF-κB [44],[45]. To determine whether M013 might also regulate the secretion of other pro-inflammatory molecules under NF-κB control, human THP-1 cells were infected with either wild-type (WT) MYXV or vMyxM013-KO virus and the cell supernatants were harvested at various time post infection to measure various indicator cytokines and chemokines by ELISA.

**Figure 1:**
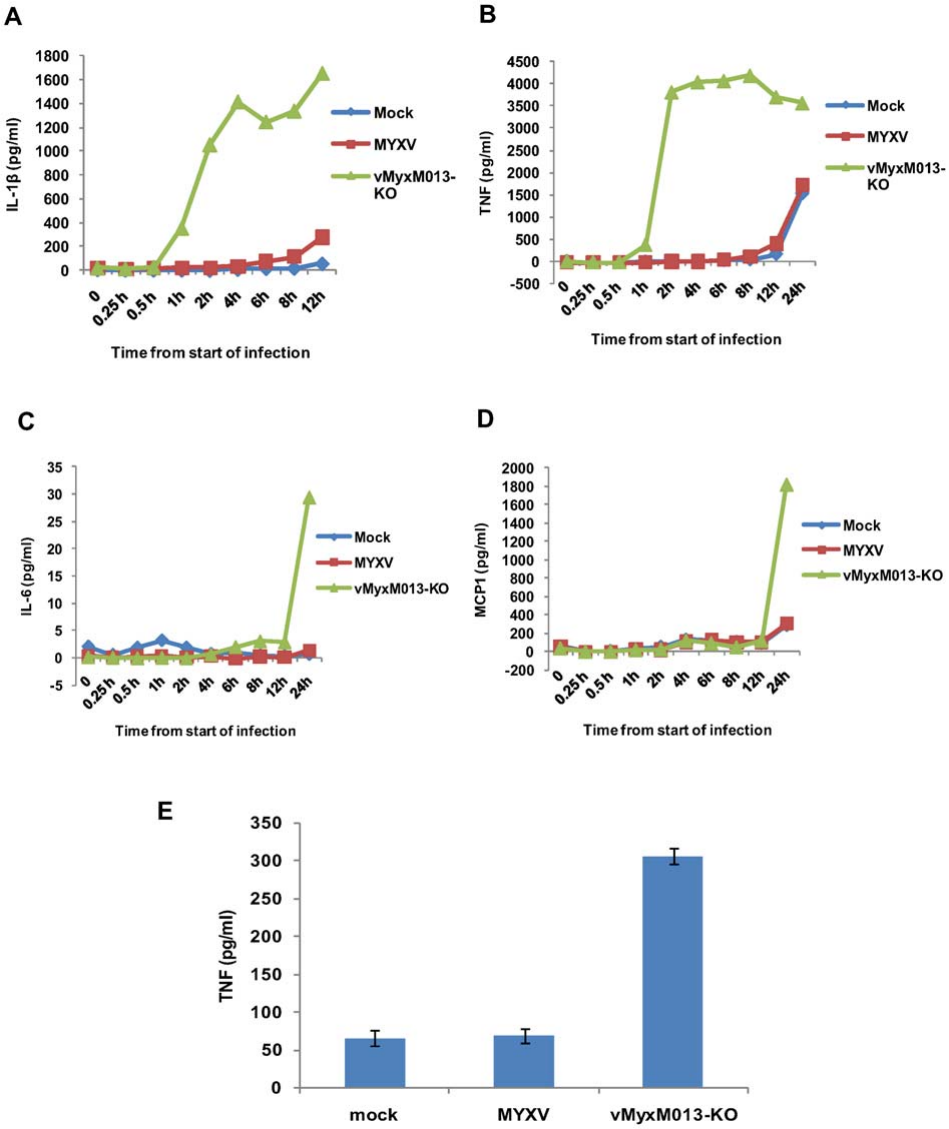
Kinetics of cytokine secretion in virus-infected THP-1 cells *in vitro* and *in vivo*. THP-1 cells were differentiated for 12–18 h with PMA and then infected with MYXV or vMyxM013-KO viruses, at MOI of 3. Cell supernatants were collected at indicated time points to measure secretion of A) IL-1β, B) TNF, C) IL-6 and D) MCP-1 by ELISA. E) Level of human TNF secretion from virus infected THP-1 tumor xenografts implanted in NOD/SCID mice by s.c injection. Virus was injected intratumorally after 7 days of implantation and tissues were collected after 48 h of virus injection. TNF was detected by ELISA.

The THP-1 cells were first treated with PMA to promote monocytic differentiation and then infected with the test viruses. When checked for virus replication in THP-1 cells both the test viruses were substantially compromised in production of progeny virions (data not shown). As a positive control, THP-1 cells were treated with LPS, a known TLR-mediated activator of NF-κB and inducer of many pro-inflammatory cytokines. In order to monitor early inflammatory cytokine responses, the supernatants were collected starting from 15 minutes after addition of virus to the cells. As shown in Fig. 1A, infection of THP-1 cells with vMyxM013-KO virus significantly increased IL-1β secretion within one hour, compared to control MYXV, indicating that the vMyxM013-KO virus was uniquely unable to block the inflammasome/caspase 1-mediated response to the virus infection. When tested for induction of an NF-κB controlled cytokine such as TNF, this cytokine level was also significantly increased within one hour, specifically in case of vMyxM013-KO virus infection but not WT-MYXV (Fig. 1B). The secretion levels of both cytokines quickly rose and co-ordinantly reached a maximum within 2 to 4 hours of infection. LPS treatment of uninfected THP-1 cells also co-induced the secretion of IL-1β and TNF to comparable levels (data not shown), suggesting that infection with vMyxM013-KO virus was as comparably robust at inducing these two cytokines as the triggering of TLR4 activation with LPS. To assess whether infection with the M013L-minus variant of MYXV induced broader effects on other pro-inflammatory cytokines, the infected THP-1 cell supernatants were also tested for IL-6, IL-12 and the CC chemokine MCP-1 by ELISA. Compared to TNF and IL-1β, the secretion of IL-6 and MCP-1 by THP-1 cells infected with vMyxM013-KO virus was first detected much later, and these were only measurable after 12 hours post infection (Fig. 1C and D). The second wave of vMyxM013-KO virus-mediated cytokine induction might be the effect of other cytokines like TNF or IL-1β. However, the control WT-MYXV that expresses M013 at this time point induced only minimal secretion of any of these cytokines. In case of LPS treatment of THP-1 cells, the induction of IL-6 and MCP-1 also began after a delay, but was first detectable after 8 hours of treatment (data not shown). Also, we did not observe any detectable induction of IL-12 and type I interferon (IFNα and IFNβ) secretion following infection of THP-1 cells with either MYXV or vMyxM013-KO viruses (data not shown). Analysis of the cytokine mRNA levels using real-time PCR revealed a similar induction of cytokine mRNA levels following infection of THP-1 cells with vMyxM013-KO (data not shown). These results indicate that, in addition to promoting inflammasome activation, infection of THP-1 cells with the M013-knockout MYXV also specifically triggered the induction of various early pro-inflammatory cytokines and chemokines that are regulated by NF-κB.

We next examined whether vMyxM013-KO virus can also induce comparable pro-inflammatory cytokine production *in vivo*, using THP-1 derived monocytic tumors established by engraftment into immunodeficient NOD/SCID mice. MYXV is currently being developed as an oncolytic therapeutic treatment for human cancer, and the WT virus replicates similarly within xenografted human tumor tissues *in situ* as it does within normal rabbit tissues [46]. Thus, to model MYXV behavior within susceptible tissues for which the appropriate cytokine reagents are currently available, engrafted THP-1 cells were used to generate monocytic tumors in NOD-SCID mice, which were first detected at 6 days after subcutaneous injection on the left hind leg. At this point, samples of either MYXV or vMyxM013-KO virus were injected intra-tumorally on day 7 and tissues were collected after 48 h post-virus injection for cytokine induction measurements. Significantly, we observed that tumors infected with vMyxM013-KO virus, but not WT-MYXV or mock PBS-injected control tumors, selectively induced significant levels of human TNF (Fig. 1E). However, we were not able to detect newly induced human IL-1β from any of these tissue samples (data not shown). Due to the localized nature of the THP-1 tumors and the virus injection route, we also could not detect any selectively induced human cytokines in the serum samples from any of the mice at this time point. This result suggests that vMyxM013-KO virus, but not WT-MYXV, can specifically induce NF-κB mediated TNF directly within susceptible tissue sites *in vivo* as well as *in vitro*.

### Activation of early NF-κB-mediated inflammatory responses by vMyxM013-KO virus infection

**Figure 2:**
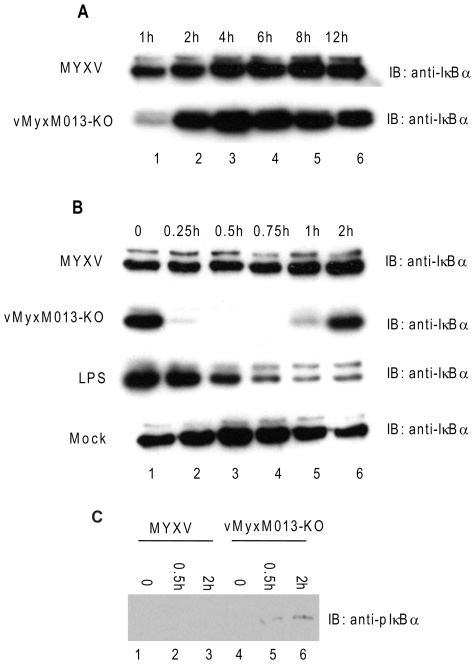
Activation of early inflammatory responses by vMyxM013-KO virus. THP-1 cells were differentiated with PMA and infected with MYXV or vMyxM013-KO viruses, at MOI of 3 or treated with LPS (1 µg/ml) for indicated time points and harvested to prepare total cell extracts. Equal amounts of protein from uninfected, infected or LPS treated cells were analyzed by Western blot using IκBα (A and B) and phospho IκBα (pIκBα) (C) antibodies.

The transcription factor NF-κB has been shown to play an essential early role in virus-induced expression of diverse pro-inflammatory cytokines, interferons and chemokines in infected cells. We next investigated how the innate NF-κB pathway was activated in response to vMyxM013-KO virus infection, which then leads to the secretion of pro-inflammatory cytokines under NF-κB control. Activation of NF-κB is usually regulated by the inhibitor IκBα, which is itself often the immediate target of viral subversion. In response to upstream stimulatory signals such as virus infection, IκBα becomes phosphorylated by cellular kinases and then is degraded by the proteasome following ubiquitination. This loss of IκBα repression then triggers the translocation of NF-κB to the nucleus to initiate target gene transcription. To determine whether increased NF-κB-driven gene transcription occurs specifically in response to vMyxM013-KO virus infection, and whether this resulted from loss of IκBα, we investigated the degradation of IκBα in virus-infected THP-1 cells by Western blot analysis. THP-1 cells were infected with WT-MYXV or vMyxM013-KO virus, harvested at different time points and the level of IκBα was assessed. By one hour post infection, we observed a transient reduction in IκBα uniquely in the cells infected with vMyxM013-KO virus, but the IκBα levels were fairly similar at later time points following either WT-MYXV or vMyxM013-KO virus infection (Fig. 2A). We then investigated the first hour post-infection in greater detail, and observed that within the first 15 minutes of vMyxM013-KO virus infection of the THP-1 cells, the cellular pool of IκBα was almost completely degraded (Fig. 2B, 2^nd^ row). However, in WT-MYXV -infected cells the level of IκBα remained essentially the same as mock infected THP-1 cells (Fig. 2B, 1^st^ and 4^th^ rows). Following this rapid degradation, the level of IκBα in the vMyxM013-KO virus infected cells then recovered and returned to normal levels within 2 hr post infection. As a comparison, we also measured the kinetics of LPS- induced degradation of IκBα in THP-1 cells (Fig. 2B, 3^rd^ row). In LPS-treated cells, the degradation of IκBα was first detected later, beginning at 30 min and the protein level continued to decrease up to 2 h post treatment, which is dramatically different from the rapid degradation induced following infection with vMyxM013-KO virus.

As the degradation of IκBα is mediated by the kinetics of its phosphorylation, we next investigated the phosphorylation state of IκBα in virus-infected THP-1 cells by Western blot analysis. The induction of phosphor-IκBα (p-IκBα) was readily detected at 0.5 and 2 hours after infection of THP-1 cells with vMyxM013-KO virus but not WT-MYXV (Fig. 2C). This suggests that degradation of IκBα in vMyxM013-KO-infected THP-1 cells is mediated by its phosphorylation, as it is by other nonviral inducers of NF-κB activation.

### Phosphorylation of IKK following vMyxM013-KO virus infection

**Figure 3:**
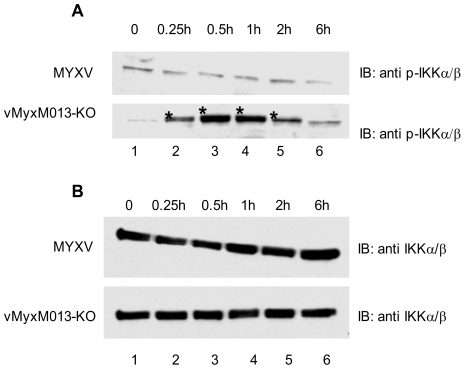
Phosphorylation of IKKα/β following virus infection. THP-1 cells were differentiated with PMA and infected with MYXV or vMyxM013-KO viruses, at MOI of 3 for indicated time points and harvested to prepare total cell extracts. Equal amounts of protein loaded in SDS-PAGE to detect proteins by Western blot using A) phospho IKKα/β and B) IKKα/β specific antibodies. The stars indicate the phospho IKK protein bands.

It is evident that vMyxM013-KO virus can induce early pro-inflammatory responses in THP-1 cells by rapidly inducing the phosphorylation and degradation of IκBα, which should trigger the nuclear translocation of NF-κB and activation of target gene transcription. We next investigated whether the multi-component IKK complex becomes activated in THP-1 cells in response to vMyxM013-KO infection and subsequently degraded the IκBα. The virus-infected THP-1 cells were harvested at different time points, and examined for IKKα/β or p-IKKα/β by Western blot analysis. In response to vMyxM013-KO virus infection, we observed a rapid elevation in the phosphorylation levels of IKKα and IKKβ, as compared to the WT-MYXV infections (Fig. 3A). The level of total IKKα/β protein was very similar when comparing the two viruses (Fig. 3B). However, the antibody used to detect the phosphorylated IKK also reacted with a non-specific protein in all the test samples. Based on this observation we conclude that early phosphorylation of IKK by vMyxM013-KO virus mediates the activation of NF-κB pathway.

### NF-κB nuclear translocation following vMyxM013-KO infection of THP-1 cells

**Figure 4:**
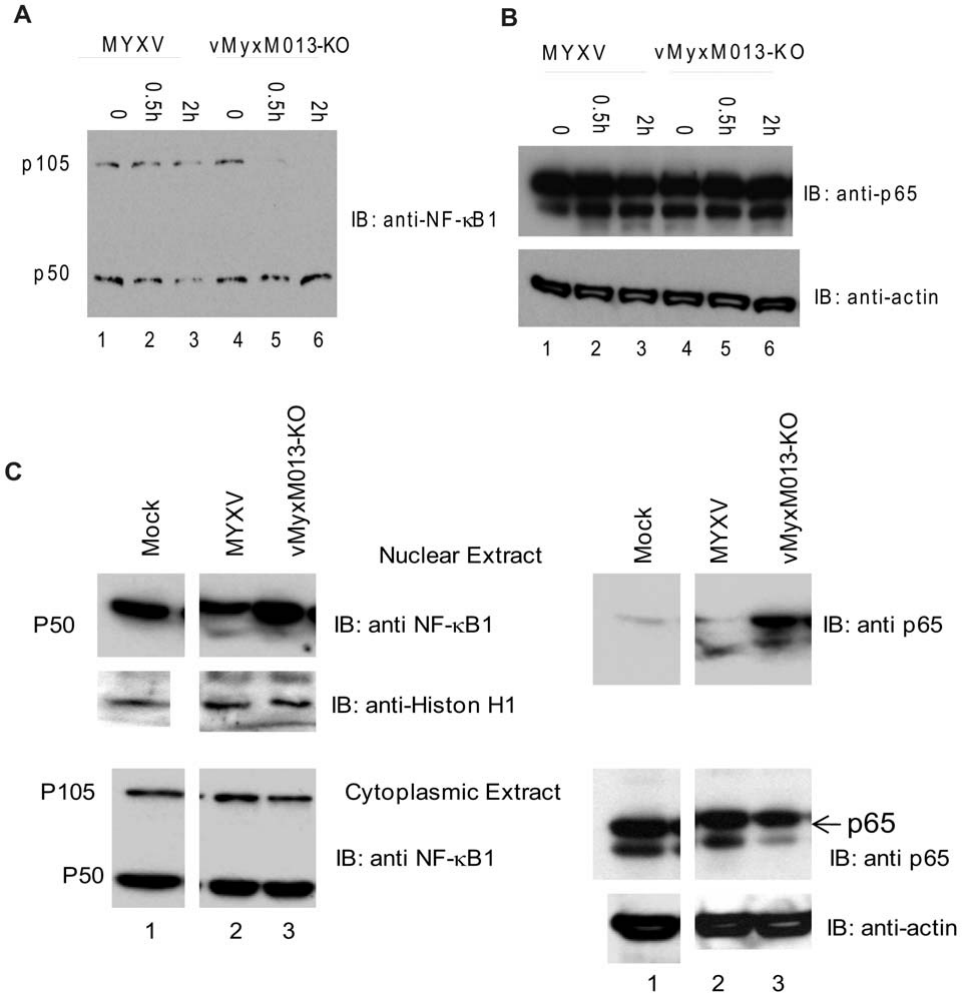
Activation of NF-κB1 and nuclear translocation of NF-κB p65 by vMyxM013-KO virus. THP-1 cells were differentiated with PMA and infected with MYXV or vMyxM013-KO viruses at MOI of 3 for indicated time points and harvested to prepare total cell extracts and nuclear and cytoplasmic extracts. Equal amount of proteins were loaded in SDS-PAGE to detect the presence of A) NF-κB1; B) NF-κB p65 in the total cell extracts and C) NF-κB1 and p65 in the nuclear (top panels) and cytoplasmic (bottom panels) extracts. The proteins were detected using specific antibodies.

We next investigated the mechanism of NF-κB-mediated activation of pro-inflammatory cytokine production in the virus-infected THP-1 cells. Among the NF-κB family members, NF-κB1 (p105 and p50) and RelA (p65) have so far been linked with virus-induced expression of pro-inflammatory target gene expression. In unstimulated cells, NF-κB1-p105 remains in the cytoplasm as a dimer with RelA/p65, c-Rel or p50. In response to activating stimuli, p105 becomes phosphorylated by kinases and is degraded to release the associated Rel or p50 subunit [47]. The p50 and p65 heterodimeric complex then translocates to the nucleus after degradation of IκBα and initiates transcription of target pro-inflammatory genes. To deduce how M013 might perturb this activation pathway, we first examined the cleavage of p105 in virus-infected THP-1 cells. The cells were infected with either WT-MYXV or vMyxM013-KO and harvested at 30 min and 2 hr for detection of protein by Western blot analysis. Infection of THP-1 cells with vMyxM013-KO virus induced the rapid degradation of NF-κB1-p105 and the NF-κB1-p50 form can be detected (Fig. 4A). However, in the WT-MYXV infected THP-1 cells, both p105 and p50 can be readily detected. The level of the p65 subunit, however, in case of both virus infections remained essentially unchanged (Fig. 4B). Thus, the expression of the M013 protein protects the p105 form of NF-κB1 from the degradation associated with the canonical NF-κB activation pathway.

In order to determine whether the RelA/p65 complex is correctly translocated to the nucleus, we prepared cytoplasmic and nuclear fractions of mock and virus-infected THP-1 cells (30 min after virus infection) and Western blot analysis was performed. In response to vMyxM013-KO virus infection, the majority of the p65 protein had migrated to the nucleus during early time points of infection (Fig. 4C, right panel). The level of p50 also increased in the nuclear fraction of vMyxM013-KO virus infection compared to the mock and WT-MYXV infection (Fig. 4C, left panel). As expected, no NF-κB1/p105 was detected in the nucleus (not shown). As loading controls, levels of histone H1 (nuclear extracts; Fig. 4C left middle panel) and actin (cytoplasmic extracts; Fig. 4C right bottom panel) were examined. These results indicate that, once THP-1 cells become activated by infection with vMyxM013-KO virus, RelA/p65 rapidly translocates to the nucleus, with kinetics similar to that triggered by many other nonviral inducers.

### vMyxM013-KO virus can induce inflammasome-dependent and NF-κB-dependent cytokine secretions independently

**Figure 5:**
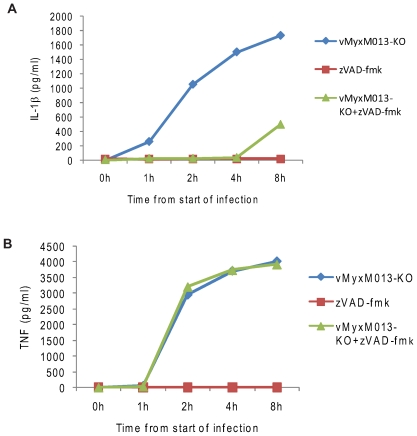
The effect of a specific caspase-1 inhibitor on cytokine secretion from virus-infected THP-1 cells. THP-1 cells were differentiated with PMA and treated with caspase-1 inhibitor zVED-fmk (50 µM) for one hour and infected with MYXV or vMyxM013-KO viruses at MOI of 3. Cell supernatants were collected at indicated time points and tested for A) IL-1β and B) TNF by ELISA kit.

In THP-1 cells, vMyxM013-KO virus infection can co-ordinantly induce inflammasome-mediated secretion of IL-1β, and IL-18, as well as NF-κB -mediated secretion of various other pro-inflammatory cytokines and chemokines. In order to determine whether these two pathways were activated independently or through common mechanism, we used a specific inhibitor of inflammasome activation to differentiate the two pathways. Inflammasome-mediated secretion of IL-1β depends on activation of caspase-1, which cleaves the pro-IL1β to produce active IL-1β for secretion. Treatment of THP-1 cells with caspase-1 inhibitor, zVAD-fmk, alone caused no induction of IL-1β but when combined with the infection with vMyxM013-KO virus resulted in the dramatic inhibition of IL-1β secretion at early time points, starting in the first hour post-infection (Fig. 5A). When the same samples were also tested for TNF secretion, we observed that inhibition of caspase-1 with this inhibitor had no effect on vMyxM013-KO virus-mediated induction of TNF (Fig. 5B). This suggests that vMyxM013-KO virus-mediated early induction of IL-1β secretion depends on inflammasome activation of caspase 1, but in case of TNF, the induction is mediated by NF-κB and is independent of caspase-1.

### vMyxM013-KO virus induces MAP kinase dependent secretion of pro-inflammatory cytokines in THP-1 cells

**Figure 6:**
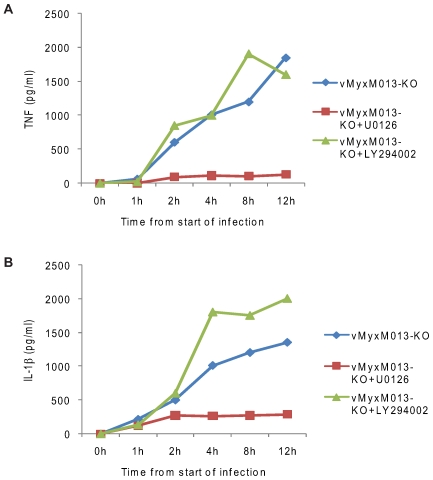
The effect of NF-κB signaling inhibitors on cytokine secretion from virus-infected THP-1 cells. THP-1 cells were differentiated with PMA and treated with ERK1/2 inhibitor U0126 (20 µM) and PI3kinase inhibitor LY294002 (20 µM) for one hour and infected with MYXV or vMyxM013-KO viruses at MOI of 3. Cell supernatants were collected at indicated time points and tested for A) TNF and B) IL-1β by ELISA kit.

In order to test which cellular signaling pathway(s) might be involved in the activation of NF-κB in response to vMyxM013-KO virus infection, we used the kinase inhibitors U0126, an ERK1/2 inhibitor and LY294002, a PI3 kinase inhibitor. THP-1 cells were pretreated with these inhibitors for one hour and infected with WT-MYXV or vMyxM013-KO viruses. The supernatants were collected at different time points after infection and tested for secreted IL-1β and TNF by ELISA. Both the kinase inhibitors had no effect on viral gene expression in this cell line when compared to the untreated cells (data not shown). Treatment of THP-1 cells with U0126 totally inhibited the vMyxM013-KO virus-induced secretion of TNF, starting from early times of infection (Fig. 6A). However, when the samples were tested for IL-1β secretion, induction of this cytokine was also inhibited and after two hours the cytokine level remains constant. This suggests that U0126 did not totally abrogate the inflammasome-mediated secretion of processed IL-1β from pre-existing stores of precursor pro-IL-1β, but did effectively inhibit the NF-κB-mediated production of precursor pro-IL-1β, which subsequently is processed by inflammasome. When tested the uninfected and infected THP-1 cells, U0126 totally inhibited the phosphorylation of ERK1/2 and IKKα/β (data not shown). The PI3 kinase inhibitor LY294002 did not inhibit the induction of either TNF or IL-1β in response to vMyxM013-KO virus infection, indicating that this pathway is not critical for induction of either cytokine in response to vMyxM013-KO virus infection (Fig. 6B). This suggests that vMyxM013-KO virus mediated activation of NF-κB is dependent on ERK1/2 kinase signaling but not the PI3 kinase pathway.

### M013 protein inhibits signaling pathways leading to activation of NF-κB in the absence of virus infection

**Figure 7:**
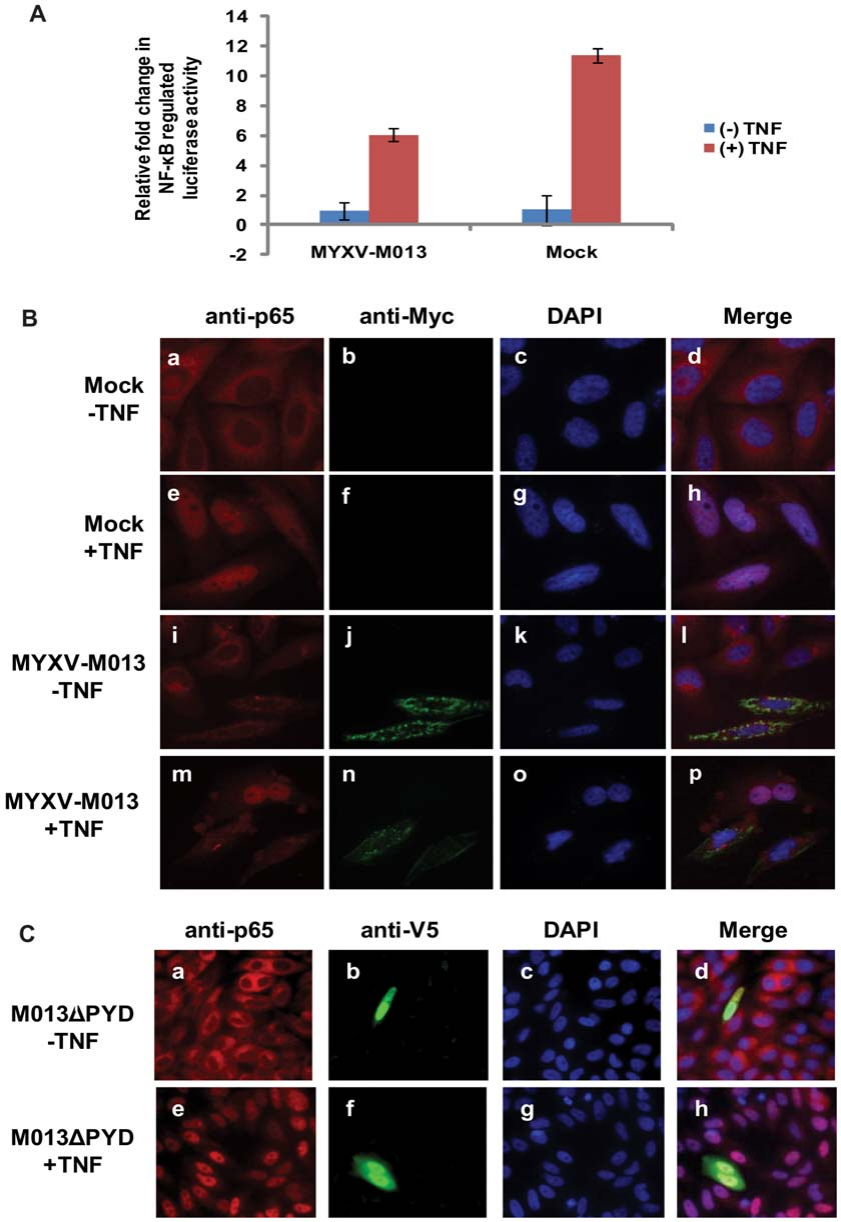
M013 protein alone suppresses NF-κB activity and nuclear translocation of p65. (A) HeLa cells were co-transfected with equal amounts of pNF-κB-Luc and tk-Renilla-Luc along with pcDNA3.1M013L Myc/His or empty vector. At 48 h post-transfection, cells were either left untreated or treated with TNF (20 ng/ml) for 6 h and then assayed for both sea pansy and firefly luciferase activity. The presented values represent the average of three independent transfections. (B) Immunofluorescence and confocal microscopy analysis demonstrating the effect of M013 on TNF-induced nuclear translocation of NF-κB/p65 in HeLa cells. Mock (a–h) or M013L (i–p) transfected HeLa cells were labeled in the absence (a–d and i–l) or presence (e–h and m–p) of TNF treatment. (C) Immunofluorescence and confocal microscopy analysis demonstrating the effect of M013ΔPYD on TNF-induced translocation of NF-κB/p65 in HeLa cells. M013ΔPYD transfected HeLa cells were labeled in the absence (a–d) or presence (e–h) of TNF treatment.

To investigate the direct effects of MYXV M013 protein on NF-κB activation, the expression of the Firefly luciferase reporter gene linked to a NF-κB dependent promoter was used to quantify induction of the NF-κB signaling pathway. As an internal control, the expression of a co-transfected Renilla luciferase gene driven by a constitutively active promoter (thymidine kinase, tk promoter) was also examined. HeLa cells were co-transfected with the plasmids expressing the reporter genes and M013L (under CMV promoter) and then the cells were stimulated with TNF for induction of NF-κB-dependent reporter gene expression. The Firefly/Renilla Luciferase expression ratio was increased significantly (about 12 fold) upon stimulation with TNF but this level was reduced (about 50%) in the presence of transfected M013L (Fig. 7A). Even with the caveats of incomplete transfection efficiencies, these results demonstrated that PYD-containing M013 protein specifically inhibits NF-κB-regulated gene expression even in the absence of any other MYXV gene products. This result is in contrast to another report claiming that a related PYD-containing poxviral protein from Shope fibroma virus, called S013, induced the activation of NF-κB activity in a transient transfection assay [48].

We next tested whether the expressed M013 protein inhibited the nuclear translocation of p65/RelA after treatment with TNF. HeLa cells were mock transfected or transiently transfected with an expression plasmid that expressed Myc-His tagged M013 protein. Immunostaining of cells using anti-p65 antibody identified the major location of this protein in the cytosol (Fig. 7B, panels a–d), however, when the cells were induced with TNF, the p65 protein efficiently translocated to the nucleus (Fig. 7B, panel e–h). In the absence of TNF stimulation, Myc-tagged M013 protein was located mostly in the cytoplasm and formed characteristic punctate bodies in the expressing cells (Fig. 7B, panels i–l). The cells which expressed M013 protein uniquely exhibited the property that TNF-induced nuclear translocation of p65/RelA was blocked (Fig. 7B, panels m–p), suggesting that the direct inhibitory effect of M013 protein on NF-κB relocation to the nucleus. The ability of M013 to inhibit the TNF-induced nuclear translocation of p65/RelA was further confirmed using a construct lacking the PYD (Fig. 7C, panels a–h). These results suggest that M013 protein in the absence of other viral proteins can inhibit NF-κB-mediated gene transcription by blocking the translocation of NF-κB p65 to the nucleus.

### M013 protein physically interacts with NF-κB1 p105

**Figure 8:**
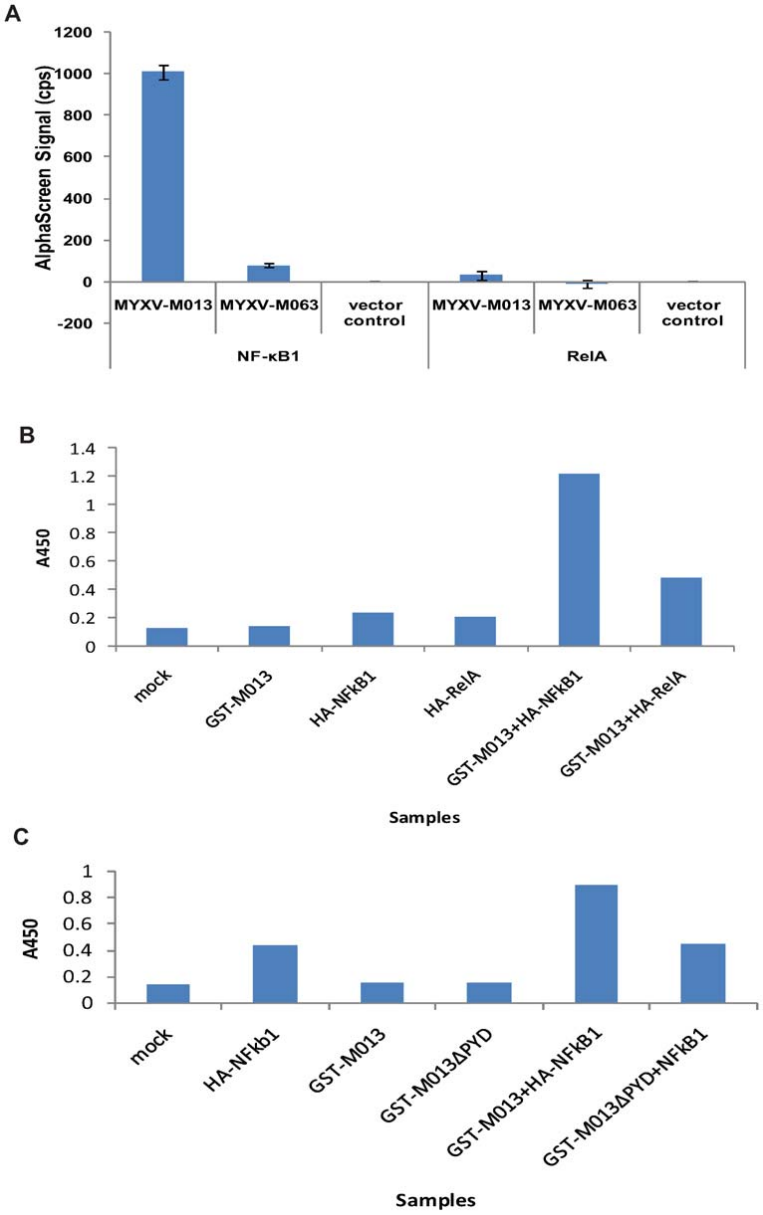
M013 protein interacts with NF-κB1 protein. A) BSR-T7/5 cells were co-transfected with the pcDNA3.1Myc/His plasmid expressing Myc/His-tagged-NF-κB1 or RelA along with the pANT7_cGST plasmid expressing GST-tagged-M013, M063 or an empty pANT7_cGST plasmid. Following transfection, cell lysates were subjected to the Alphascreen protein-protein interaction assay using Nickel-coated donor beads and anti-GST-coated acceptor beads. The presented results represent the average of three independent transfections. Using *in vitro* TNT reactions GST-tagged M013 B) or M013ΔPYD C) and HA-tagged NF-κB1 or RelA were expressed alone or co-expressed from appropriate plasmids. The expressed proteins were bound to the anti-GST antibody coated 384 well plates. After washing the bound protein complexes were detected using anti-HA antibody and a HRP-coated secondary antibody. The binding of secondary antibody was detected by applying TMB substrate. The plate was then read at 450 nm using a multiwell plate reader.

To better understand how M013 protein prevents translocation of RelA/p65 to the nucleus, we examined the physical interactions between various NF-κB members and M013 protein using *in vivo* and *in vitro* protein-protein interaction methods. *In vivo* interaction was studied in transfected cells using the AlphaScreen method [41],[42]. Plasmids expressing GST-tagged M013 protein and Myc/His tagged host proteins under a T7 promoter were co-transfected in T7 polymerase-expressing BSRT7/5 cells and tested using specific AlphaScreen beads. Interaction of M013 protein with human NF-κB1 p105 protein was detected by emission flourescence whereas a control poxviral protein of a similar size (MYXV M063) or vector control (Fig. 8A) did not. Using the same method, however, we did not detect any interaction between M013 and RelA/p65. The interaction of GST-tagged M013 and Myc/His tagged NF-κB1 p105 was also confirmed by GST pulldown assay after co-expression of the corresponding plasmids in the BSRT7/5 cells (data not shown).

The protein/protein interaction between M013 and NF-κB1-p105 following expression in reticulocyte lysates was also confirmed using an ELISA method. GST-tagged M013 protein and HA-tagged host proteins were individually (as control) or co-expressed using the *in vitro* TNT expression system. The GST-tagged M013 viral protein was bound to the ELISA plate coated with anti-GST antibodies and the interacting HA-tagged host protein was detected using HRP conjugated anti-HA antibody. Using this method, we could detect the interaction of M013 with NF-κB1-p105, while RelA produced essentially no signal above background (Fig. 8B). In addition, the pyrin domain was specifically shown to be required for this interaction because when this domain was deleted from M013, no binding with NF-kB1 was detected (Fig. 8C). This confirmed that M013 protein can associate directly with NF-κB1-p105 in the absence of any other viral proteins.

## Discussion

The MYXV-encoded M013 protein was first characterized as a functional member of the cellular PYRIN domain (PYD)-containing protein family that regulates the function of host inflammasome complex [33]. The PYD domain is one of four subfamilies of the death-domain fold superfamily of evolutionary conserved protein-protein interaction domain containing proteins [34],[49]. PYD-containing proteins mediate homotypic and heterotypic interactions among family members and even proteins with different domains, from other families. These diverse interactions allow PYD-containing proteins to be associated with cellular processes like apoptosis, immunity, inflammation, differentiation and proliferation [34]. In humans, at least 23 PYD containing proteins have been identified to date that fall in four groups: 1- receptor proteins or pathogen recognition receptors (PRRs), that recognize pathogen associated molecular patterns (PAMPs) known as PAN, NALP (NACHT, LRR and PYD containing proteins), PYPAF, NOD (nucleotide-binding oligomerization domain), Caterpiller and NLR (NOD-like receptors) proteins [35],[50],[51]; 2- adapter proteins like ASC-1 [52],[53]; 3-regulatory proteins, for example cellular PYD-only proteins (cPOPs) cPOP1 and cPOP2 [44],[45],[54] and, 4- IFN-inducible proteins known as 200-amino-acid repeat (HIN-200) gene family [55].

The PYD-containing host proteins are predominantly expressed in leukocytes and tissue sentinel cells and are integrated with pathogen sensors that trigger the innate immune responses. The common adapter protein ASC-1 contains bifunctional domains PYD and CARD (C-terminal caspase-recruitment domain) which interacts with the PYD-containing NALP proteins and CARD-containing caspase-1, respectively, to activate pro-caspase-1 in the NALP3-inflammasome complex [53]. ASC-1 also regulates apoptosis and NF-κB signaling responses by interaction with multiple other proteins in those pathways [52]. ASC-1, in turn, is regulated by cellular and viral regulatory proteins, collectively called POPs [33],[44],[45],[48],[54],[56]. The cellular regulatory proteins, cPOP1 and cPOP2, control the function of adapter protein ASC-1 by mediating PYD-PYD interactions. In addition to ASC-1, cPOPs also can inhibit NF-κB activation [44],[57]. M013 protein of MYXV is an example of a functional viral POP (vPOP). M013 interacts directly with ASC-1 protein and modulates caspase-1 activation and IL-1β/IL-18 processing in cultured monocyte-derived cells, such as THP-1 cells, and in infected rabbit tissues [33]. A closely related vPOP protein from Shope Fibroma virus was also shown to block the activation of pro-caspase-1 in transient transfection assay and subsequent processing of pro-IL-1β [48]. Furthermore, infection of THP-1 cells with vMyxM013-KO virus induced the activation of caspase-1 and processing and secretion of pro-inflammatory cytokines IL-1β and IL-18 [33]. Here, we demonstrate that vMyxM013-KO infection of THP-1 cells also induces other cytokines and chemokines (TNF, IL-6, and MCP-1) that are regulated by the NF-κB pathway, in addition to the cytokines controlled by the caspase 1/inflammasome complex.

Both cPOP1 and cPOP2 have been shown to also exert inhibitory properties for NF-κB signaling [44],[45]. On the other hand, PYD-containing pathogen receptors (PRRs) have diverse effects on the function of NF-κB. Some of these are reported to have NF-κB-mediated pro-inflammatory roles such as PYPAF1/NALP3 [58], whereas others such as PAN2/PYPAF4 and PAN1/PYPAF2 inhibited NF-κB [59]. The vMyxM013-KO virus-mediated activation of NF-κB reported here suggests that the M013 vPOP protein has a co-inhibitory role against both the inflammasome complex and cellular NF-κB signaling, and binds distinct protein targets from each pathway, namely ASC-1 and NF-κB1.

One of the novel findings in this study is the rapid induction of NF-κB-mediated inflammatory responses against vMyxM013-KO virus, which is repressed by the WT-MYXV. Infection of THP-1 cells with vMyxM013-KO virus induced the formation of NF-κB p50/p65 complex, which migrates to the nucleus to induce the transcription of pro-inflammatory mediators. Indeed, transient expression of M013 protein alone inhibited the TNF-induced NF-κB activation by blocking the translocation of p65 to the nucleus. The host cPOP2 also blocks the NF-κB activation in the same fashion [45]. Although in case of POP2, the mechanism of this inhibition is not known, POP1 interacts with IKKα, which likely affects the downstream phosphorylation of IκBα [44]. M013 protein, on the other hand, interacts directly with NF-κB1/p105 and we postulate that this complex prevents the subsequent nuclear translocation of p50/p65 complex at a point downstream of IKK activation. For example, the interaction between M013 and NF-κB1 might interfere with the degradation of NF-κB1/p105, and therefore prevent the release of active p50 and the formation of the active p65/p50 heterodimer that subsequently translocates to the nucleus. Thus, the vMyx-M013KO-induced phosphorylation of IKK and degradation of IκBα raises the possibility that deletion of MYXV-M013 from MYXV triggers change(s) in the cellular sensing and/or responses to virus infection such that it hinders the ability of the other viral gene products to prevent the degradation of IκBα. Very recently, it has been shown that a different family of ankyrin-repeat containing viral proteins, encoded by orthpoxviruses also interacts with NF-κB1/p105 and inhibits the activation of the NF-κB pathway [60]. This suggests that the NF-κB1/p105 is a more common target for viral modulation than previously appreciated.

It is not yet determined which protein domain of NF-κB1 is involved in interaction with the M013 vPOP. Since NF-κB1 itself lacks any PYD domain, the interaction is predicted to be heterotypic, which has been previously observed in case of other PYD-containing proteins [52]. It is also possible that the PYD of M013 may interact with multiple signaling molecules in the NF-κB pathway, based on the reported ability of PYD to enter into heteromeric complexes and as demonstrated for ASC-1 [52]. It is also probable that MYXV encodes multiple proteins to inhibit the NF-κB pathway at different stages, as reported in the case of VACV and MOCV [22],[23],[24],[25],[27]. The MYXV-encoded ankyrin repeats (ANKs) containing protein M150 has previously been proposed to be regulator of NF-κB function [61]. Also called myxoma nuclear factor (MNF), M150 protein of MYXV migrated to the nucleus in response to TNF treatment and co-localized with NF-κB p65 [61] but to date no interacting host protein has yet been reported for MNF/M150.

From our observations we propose that the single PYD-containing vPOP protein M013 is able to simultaneously associate with components from both the inflammasome complex (i.e. ASC-1) and NF-κB signaling pathway (i.e. NF-κB1) to efficiently downregulate both these arms of the innate cellular pro-inflammatory responses to virus infection. In the cellular context, PYD-containing proteins are very important in diverse processes, as mutations in the part of the genome that encodes certain cellular PYD family members are connected with hereditary diseases such as familial Mediterranean fever (FMF), familial cold autoinflammatory syndrome (FCAS), Muckle-Wells syndrome (MWS), and Neonatal-onset multisystem inflammatory disease (NOMID) [62],[63]. For example, the vast majority of FMF-associated mutations are located in the C-terminal B30.2 (SPRY) domain of the *MEFV* (Mediterranean Fever) gene-encoded protein product pyrin. Any of these mutations in fact disrupt the regulatory role of pyrin in caspase-1 activation and IL-1β production [64],[65]. In case of MYXV, the PYD domain of the viral protein is important for the pathogenicity of the virus *in vivo* and the lack of M013 expression induced a rapid early inflammatory cytokine response (featuring caspase-1-dependent induction of IL-1β and IL-18) in the infected host lesions [33].

The successful co-inhibition of both inflammasome activation and NF-κB -mediated inflammatory signaling responses by a single viral immunomodulator vPOP, M013, represents a unique bi-functional strategy deployed by poxviruses to dampen multiple early innate immune responses to the infecting virus. Next, it is critical to better understand the mediator used by the host cells to sense the infecting vMyxM013-KO virus to rapidly induce both the inflammasome and NF-κB pro-inflammatory responses and how the M013 vPOP protein intercepts these signals.
